# Disentangling constraints using viability evolution principles in integrative modeling of macromolecular assemblies

**DOI:** 10.1038/s41598-017-00266-w

**Published:** 2017-03-22

**Authors:** Giorgio Tamò, Andrea Maesani, Sylvain Träger, Matteo T. Degiacomi, Dario Floreano, Matteo Dal Peraro

**Affiliations:** 1 Laboratory for Biomolecular Modeling, Institute of Bioengineering, School of Life Sciences, École Polytechnique Fédérale de Lausanne, Lausanne, CH-1015 Switzerland; 2 Swiss Institute of Bioinformatics (SIB), Lausanne, CH-1015 Switzerland; 3 Laboratory of Intelligent Systems, Institute of Microengineering, École Polytechnique Fédérale de Lausanne, Lausanne, CH-1015 Switzerland; 4 Chemistry Research Laboratory, Department of Chemistry, University of Oxford, Oxford, UK

## Abstract

Predicting the structure of large molecular assemblies remains a challenging task in structural biology when using integrative modeling approaches. One of the main issues stems from the treatment of heterogeneous experimental data used to predict the architecture of native complexes. We propose a new method, applied here for the first time to a set of symmetrical complexes, based on evolutionary computation that treats every available experimental input independently, bypassing the need to balance weight components assigned to aggregated fitness functions during optimization.

## Introduction

Macromolecular assemblies are of paramount importance for the functioning of biological cells. Due to their size and complexity, using traditional experimental methods to elucidate their structure and dynamics remains to date a daunting task and a major challenge. Nevertheless, if high-resolution structures of subunits as well as experimental low-resolution information describing their mode of assembly are available (e.g., describing residue contacts between protein subunits), these can be used to assemble the subunits into their native complex. Integrative modeling (IM)^1–3^ is an *in silico* approach that integrates this experimental information with empirical energy potentials, as found in molecular force fields^4^, to generate candidate model assemblies. Along with the recent advances in structural biology^5, 6^, IM has gained importance by successfully predicting several large molecular assemblies from their isolated subunit components^4, 7–10^.

The energetic and experimental components are usually aggregated into a fitness function that describes the quality of the candidate assemblies and that is minimized by a stochastic search^4, 11–13^. In order to balance the contribution of the components, relative weights must be assigned^8, 11^. The determination of optimal weights, besides being a tedious and computationally expensive process, can heavily influence assembly predictions^14, 15^. A further and more general issue consists in the fact that a unique fitness function, notwithstanding its correct component balancing, is often inadequate to select the best candidate assemblies generated by IM protocols^1^. This inaccuracy results from the difficulty to correlate the quality of the candidate solutions to canonical scoring terms^16^.

Here we report a widely applicable IM protocol based on an evolutionary method that does not require the individual weighting of the fitness function components. The proposed protocol is based on a novel evolutionary method^17^, where candidate solutions can survive and reproduce if they satisfy a set of viability criteria defined on the problem objectives and constraints. In particular, we adopt a variation of the viability method, named memetic Viability Evolution (mViE)^18^, which maintains and recombines multiple sub-population in order to optimize the balance between local and global search and has been shown to outperform several state-of-the-art methods on a standard benchmark suite of constrained optimization problems^18, 19^ (see Methods). Therefore, rather than mixing objectives and constraints in a single fitness function, this method modifies independently the viability criteria for each objective and constraint during evolution, driving thus the solutions towards desired regions of the search space. Practically, this allows for a natural partitioning of the fitness function components. In the case of macromolecular assembly prediction, this means that fitness function components can be treated independently as objectives and inequalities representing constraints on the search space. The resulting assembly protocol based on mViE is featured as an extension of our protein assembly framework *pow* ^*er*^ (http://lbm.epfl.ch/resources)^7, 11^, and it is benchmarked here on an extended set of known symmetrical assemblies.

## Results and Discussion

### Viability evolution applied to assembly prediction

**Figure 1:**
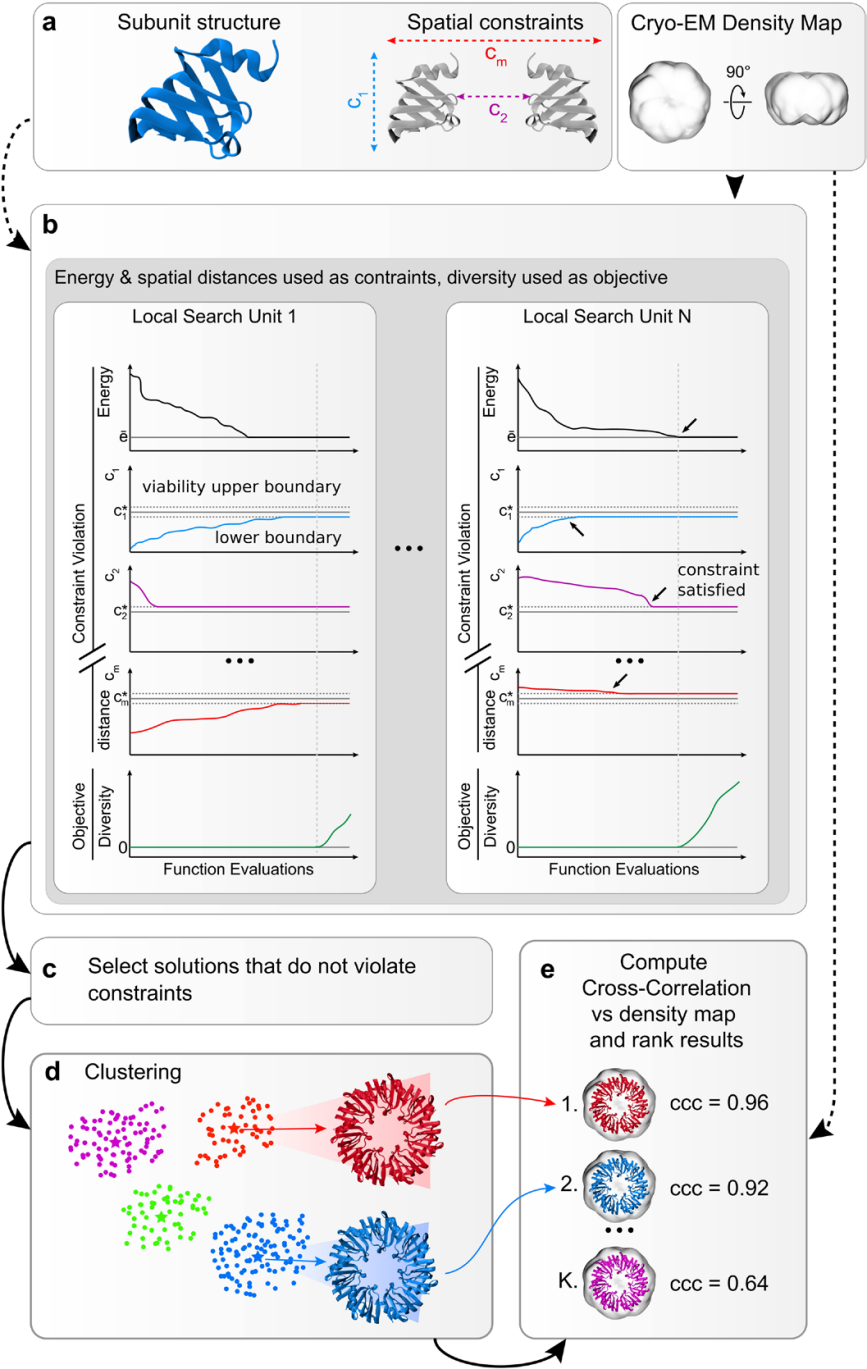
Assembly prediction using mViE algorithm. (**a**) The protocol requires as input structures at atomic resolution of the subunits forming the assembly, a set of spatial constrains obtained by experiments that characterize the connectivity of the complex, and/or volumetric density maps providing information about the general complex architecture. (**b**) mViE uses a population of multiple search units that try to independently minimize each constraint violation defined either as energy for the coarse energy potential or as C_{1, 2, … m}_ for the residue spatial distances. Once a search unit discovers assemblies that satisfy all the constraints, i.e. that are within the viability upper and lower boundaries defined on the constraints (black arrows on left panel), it tries to discover assemblies that maximize the diversity with respect to the assemblies predicted by the other search units. This is performed by favoring solutions that are at a larger Euclidean distance in the search space with respect to other local search units. (**c**) Candidate assemblies that do not violate the constraints are (**d)** hierarchically clustered and the clusters centroids are extracted. (**e**) The centroids are ranked on their cross-correlation coefficient (*ccc*) computed against the provided density map and the top-ranked predictions are returned to the user. Notice that density maps can be used *a priori* as objective or *a posteriori* for ranking.

**Figure 2:**
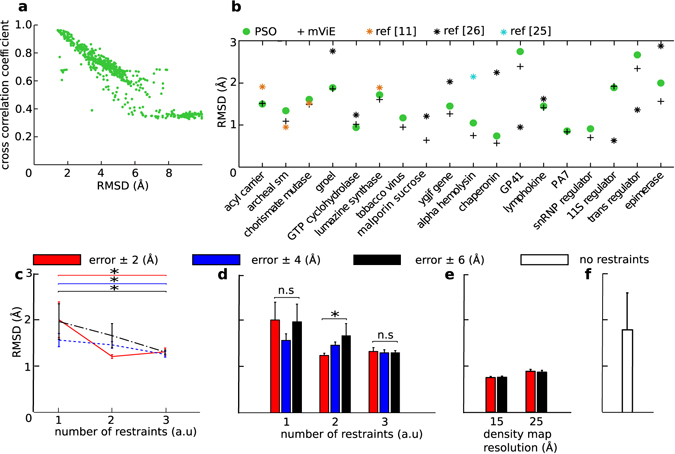
Performance assessment of mViE. (**a**) Cross-correlation-coefficient (*ccc*) and backbone RMSD landscape of the lymphokine assembly models is shown as representative result. (**b**) Performance comparison between mViE, PSO, and the best candidate assemblies of related protein predictions as reported in the literature^11, 25, 26^ on 18 symmetrical protein assembly predictions. (**c**) Effect of number of geometric spatial constraints on the quality of candidate assemblies returned by mViE on the GTP-cyclohydrolase homo 5-mer assembly problem (used as representative case, see Supplementary Fig. S2 for extended data). Here the spatial constraints and potential energy were used as constraints on the search space, population diversity as objective and density map ranking as a post-processing step. (**d**) Effect of the quality of spatial constraints (measured as error ± Å) on the quality of candidate assemblies returned by mViE on the GTP-cyclohydrolase homo 5-mer assembly problem. The assembly conditions were the same as in panel (c). (**e**) Results of mViE protocol using density map fitting during assembly of GTP-cyclohydrolase. The optimization was undertaken by using *ccc* as an objective to be maximized once the spatial and energy constraints are satisfied. (**f**) Blind docking using mViE on the GTP-cyclohydrolase assembly problem. As input were provided a density map of 15 Å and one of the homo 5-mer subunit. The *ccc* was used as objective to be maximized during the optimization. Only the energy potential was used as constraint during the optimization procedure.

We initially tested mViE on a benchmark set of symmetric assemblies where inputs are given as (i) high resolution structures of the subunits, (ii) realistic spatial constraints describing connectivity among subunits (as derived from cross-linking mass spectrometry experiments, e.g.), and/or (iii) volumetric density maps as obtained by cryo-Electron Microscopy (EM), e.g. (Fig. 1a). During the prediction of plausible assemblies (Fig. 1b), we used mViE to explore the assembly conformational space of subunits. At first, mViE attempts to find non-violating structural models, termed viable, by using the coarse energy potential as well as residue distances as viability boundaries. This is to ensure that structural models without steric clashes which satisfy the imposed distances of contacting interfacial residues are found (Fig. 1b). Then, mViE attempts to maximize, as objective, the diversity of viable solutions. These solutions are clustered, fitted into their assembly density maps provided as input^20^ and finally ranked according to a cross-correlation coefficient (*ccc*) value (Figs 1c and 2a, see Methods). Low-resolution volumetric maps or, in general, additional experimental inputs are not always available to rank the candidate solutions. Clustering algorithms can alone identify best solutions as they tend to predominantly sample regions of the search space associated to the most native-like assembly^21^. Moreover, multi-resolution energy scoring functions can be applied to assess assembly predictions on the sole basis of intermolecular contacts^22^, as for instance using recent machine learning protocols to discriminate true protein-protein interfaces from incorrect ones^23, 24^. Eventually, final clustered solutions can be further refined using more sophisticated and computationally expensive techniques.

### Performance and versatility of mViE: switching constraints and objectives

In order to assess the feasibility and performance of this new protocol, we collected 18 symmetrical assemblies for which the multimeric conformation is already known, and compared the results with previously proposed methods including a Particle Swarm Optimization (PSO) *pow* ^*er*^ ^11^, Multifit^25^ and SymmDock^26^ (see Methods and Supplementary Table S1). For each of the assembly cases, EM maps at 15 Å resolution were synthetically generated and used to rank the best candidate solutions. Ranking by density maps was more efficient in extracting the best models compared to using solely the coarse energy potential, as demonstrated by a better correlation between relative model rmsd and *ccc* value (Fig. 2a and Supplementary Fig. S1 and Table S2). Remarkably, mViE is on par or better (in 13 out 18 protein prediction problems, mViE returned solutions with lower RMSD values than other prediction protocols, Fig. 2b and Supplementary Table S3) than existing IM protocols while it did not require an aggregated fitness function and, most importantly, the *a priori* identification of weights for the fitness components.

In the previous assessment, density map fitting was used as a post-processing step to rank the best assemblies returned by mViE. However, taking advantage of the flexibility of mViE, this kind of input can be used upfront as objective, while the interfacial residue distances and energy potential are still used as constraints on the search space. When applied to a selected set of complexes (i.e., GroEL, GTP-cyclohydrolase, and lymphokine complexes, see Online Methods) we could observe a significant improvement in term of solution quality (P < 0.05, Wilcoxon-Rank-Sum-Test, Fig. 2c–e and Supplementary Fig. S2), demonstrating the flexibility of mViE in effortlessly handling additional and diverse components concurring to global fitness. Along the same lines, mViE was further tested on the problem of blindly docking protein subunits into density maps alone, i.e., without any additional distance constraint. We thus used solely the *ccc* value as objective to be maximized upon satisfaction of the energy potential as constraint. We found that mViE was able to successfully assemble complexes inside their density map for all the three selected assemblies (Cα RMSD < 2.5 Å, Fig. 2f and Supplementary Fig. S2).

To better understand how geometric constraints affect the outcome of mViE, a comprehensive benchmark was performed on the same selected targets (see Methods and Supplementary Fig. S3). We found that increasing the number of distance constraints improved the quality of solutions more directly than changing the accuracy of the constraints, (P < 0.05, Jonckeere-Terpstra-Test, Fig. 2c,d and Supplementary Fig. S2). Changing density map resolution on the other hand seemed to have little effect on solutions quality. Specifically, the best solutions extracted with density maps at a resolution of 15 Å were not different (P > 0.05, Wilcoxon-Rank-Sum-Test, Fig. 2e and Supplementary Fig. S2) than those ranked with a resolution of 25 Å.

### Assessing the quality of experimental constraints

**Figure 3:**
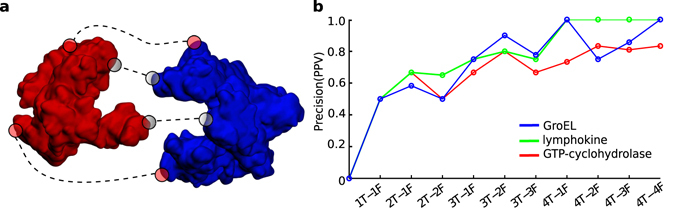
Detection of wrong constraints using mViE. (**a**) Protein-protein interaction example where 4 interfacial residue contacts are used as inputs, in this case 2 of these constraints are correct (grey colored circles) and two are erroneous (red colored circles). (**b**) Wrong constraints inclusion effect on returned model quality. The quality of the models was assessed by calculating the Precision/Positive Predictive Value [PPV = TP/(TP + FP)] representing how well good constraint (TP) were correctly satisfied in the returned models against wrongly satisfied “bad” constraints (FP). On the x-axis, the 1T-1F label means that 1 correct and 1 wrong spatial constraints were used during the optimization process.

While the assembly problems described so far were ideal cases where experimental constraints used as input were in fact correct, in reality ambiguity in the experimental constraints can arise, for example due to intrinsic limitations of techniques or multiple conformational states of the assembly. Unfortunately, in these cases correct and erroneous experimental constraints are difficult to discriminate *a priori* and are both used, possibly leading to incorrect models. A promising approach to solve this challenging problem has been recently proposed within a Bayesian framework^8, 9, 14^. Within our scheme, mViE search units naturally return solutions that maximize the number of constraints satisfied. Thus, if we realistically assume that the number of incorrect experimentally derived constraints never outnumbers the number of correct constraints, we can expect mViE to return solutions that satisfy a greater majority of correct constraints. To test this hypothesis, we synthetically increased the number of wrong constraints (see Methods and Supplementary Table S4), and could observe that for all the 3 selected cases, mViE was eventually able to return models satisfying a greater majority of correct constraints (Fig. 3a,b). Therefore, when dealing with a large amount of experimental constraints, mViE represents a promising method to effectively maximize the probability to select native structural features while discarding erroneous or inconsistent experimental inputs during model prediction.

### Predicting the interface of the PhoQ periplasmic sensor

**Figure 4:**
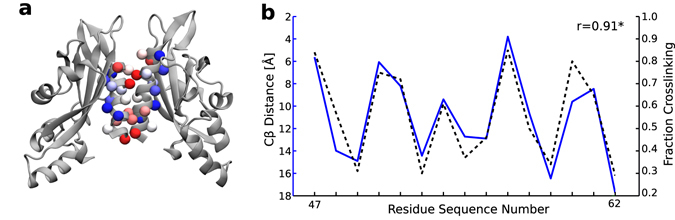
Assembly of a periplasmic sensor. (**a**) Best model of the PhoQ periplasmic sensor obtained by mViE. The colors of residue Cβ atoms represent the degree of crosslinking from highly (red) to poorly (blue) crosslinked. (**b**) Fraction crosslinking vs. Cβ distance for the best structural model of the PhoQ periplasmic sensor.

We finally applied mViE to the assembly of the PhoQ perisplasmic sensor^27^. In order to reconstruct the biological PhoQ homo-dimer interface, we used subunit model structures of PhoQ and a set of crosslinking fractional data as experimental inputs^27^. In this case we were able to directly model the PhoQ complex as a constrained optimization problem; in particular, the energy potential and the distance between Cβ of the most highly cross-linked residues were used as constraints on the search space (see Methods). Since the distance between disulfide cross-linked residues is correlated to their degree of cross-linking^27^, we used as objective the Pearson correlation coefficient (*R*) between the fractional cross-linking profiles and the Cβ distances between cross-linked residues. The predicted PhoQ interface was in agreement with the crosslinking profile obtained experimentally (Pearson *R* = 0.91, p < 0.05, Fig. 4a,b). Compared to the original study where 774,165 models were generated^27^ to find the optimal solutions, less than 3000 models were generated with mViE.

## Conclusion

In conclusion, here we described and experimentally validated a new viability evolutionary algorithm that shows great flexibility and efficiency when applied to integrative modeling problems. This new method enables the independent treatment of experimental data without the need of identifying weights and combination of individual constraints into an aggregated fitness function. The method can thus handle a large and heterogeneous variety of experimental inputs related to molecular assemblies, and assess at the same time the quality of the predicted models and experimental inputs used in model prediction. This method could be extended to more challenging and general cases such as the prediction of large non-symmetric heteromultimeric complexes. The absence of symmetry would increase computational demand due to a larger number of dimensions to be explored, likely requiring more spatial constraints to help convergence toward predicted assemblies consistent with the initial inputs.

## Methods and Materials

### *pow*^*er*^ framework for macromolecular assembly

Our software for predicting assembly structures, *pow* ^*er*^ (http://lbm.epfl.ch/resources) is originally based on a particles swarm optimization (PSO) algorithm called *PSO-kar*, featuring a heuristic approach to avoid premature convergence^11^. *pow* ^*er*^ has demonstrated its ability to predict symmetric assemblies with varying stoichiometries and shapes^7, 11^. *pow* ^*er*^ uses the symmetry of assemblies by optimizing the roto-translational parameters of only one subunit. The whole complex can be modeled simply from circular rotations of that single subunit. These parameters consist of [*α*, *β*, *γ*, *x*], where *α*, *β*, *γ* are the three Eulerian angles defining the protein orientation and *x* the radius of the symmetric assembly. In its earliest implementation, *pow* ^*er*^ predicts symmetric protein assemblies as an unconstrained optimization problem, where a single objective function *f(x)* has to be minimized which consists in:

1 $$f(x)={\boldsymbol{w}}\ast {E}_{phys}+(1-{\boldsymbol{w}}){E}_{data}$$

where$\,{E}_{data}=\sqrt{\sum _{i}^{n}{({o}_{i}-{t}_{i})}^{2}}$ combines the squared sum of the differences between target and observed spatial constraints (*n* is the numbers of geometric constraints, *o*_*i*_ is the Euclidian distance of the constraint *i* observed in the assembly model and *t*_*i*_ its target distance value, as inferred from experimental data); and ${E}_{phys}=4\varepsilon [{(\frac{\sigma }{r})}^{9}-{(\frac{\sigma }{r})}^{6}]$ is a 9-6 Lennard-Jones coarse energy potential^28^ used to avoid steric clashes (*r* consists in the pairwise distance between the Cα interfacial atoms of the subunits within a distance of 12 Å, *σ* = 4.7 Å and *ε* = 1 kcal/mol). In order to balance the energy and geometric contribution inside the fitness function *f(x)*, a weight factor ***w*** = 0.2 was used^11^.

### Prediction protocol based on viability evolution

The memetic Viability Evolution (mViE) algorithm evolves a population of local search units, based on Covariance Matrix Adaptation Evolution Strategy (1 + 1)-CMA-ES^29^ enriched with techniques for adapting the covariance matrix and the step-size^19, 30^. The mViE algorithm^18^ evolves a population of (1 + 1)-CMAES local search units that are recombined using Differential Evolution (DE)^31^ operators in order to perform a global search. A scheduler switches between advancing local search units and/or recombining them. This method has been shown to outperform several state-of-the-art methods on a standard benchmark suite of constrained optimization problems^18^.

mViE was incorporated into the *pow* ^*er*^ framework in order to model the prediction of symmetric assemblies as a constrained optimization problem without aggregating geometric distances (*E*_*data*_) and energy potential (*E*_*phys*_) in the same fitness function. In this case *E*_*data*_ was used as a constraint on the search space to guide the assembly of the subunits. During optimization, the energy potential (*E*_*phys*_) can be used in two different ways. On one hand, *E*_*phys*_ can be used as an objective to be minimized once the *E*_*data*_, used as constraints, have been satisfied in the form of:

2 $$min\,{E}_{phys},{s}{\rm{.}}{t}{\rm{.}}\{{l}_{i}\le {t}_{i}\le {u}_{i},i=1,2,\ldots, n\}$$

where *l*_*i*_ and *u*_*i*_ are the lower and upper target boundaries of each of the *n* contacting residue distances, respectively. On the other hand, *E*_*phys*_ can be used together with *E*_*data*_ as a constraint on the search space. In this case, maintaining *E*_*phys*_ < 0 would make sure that the assemblies are not only without steric clashes but also in close proximity.

Population diversity is a measure of how diverse the population of mViE search units are from a candidate solution *x* generated any time during optimization. Maximizing this term during optimization would increase the diversity of candidate solutions by a better exploration of the feasible search space, i.e. toward regions of the search space where candidate solutions do not violate constraints. Diversity can be measured as:

3 $$diversity({x}_{t})=\,\frac{\sqrt{\sum _{i=0}^{n}{({x}_{t}-X{(i)}_{population})}^{2}}}{n}$$

where *x*_*t*_ is the parameter of a candidate solution generated at step *t* of the optimization, *X* the parameter of a mViE local search unit *i* from a population of size *n*.

In the case where *E*_*phys*_ and *E*_*data*_ are used as constraints on the search space, population diversity can be used as objective to be maximized once the candidate solutions have satisfied the above constraints in the form:

4 $$max\,diversity,{s}.{t}.\{\begin{matrix}{l}_{i}\le {t}_{i}\le {u}_{i},\,i=1,2,\ldots,n \\ {E}_{phys} < 0\end{matrix}$$

### Ranking of candidate solutions with electron density maps

Due to the fact that a large number of solutions were generated during optimization, representative structures of the best models needed to be extracted. To do so, an integrated hierarchical clustering algorithm that selects centroids of clustered solutions with respective backbone root-mean-square-deviation (C*α*-RMSD) lower than 1 Å was used. Input density maps of the reference structures were simulated using the SITUS^32^ package command *kercon*, to rank the centroid of each cluster at resolutions 15 and 25 Å.

The centroids were then independently fitted in the simulated maps using the SITUS module *colores* and ranked according to a cross-correlation coefficient (*ccc*), which described the overlap between model and map. SITUS can fit structures into density maps with resolutions as low as 30 Å thanks to an intermediate step of Laplace transformation of the map that improves shape definition^32^.

### Assessing the performance of mViE against PSO

In order to assess the performance of the mViE protocol against a previously published algorithm Particle Swarm Optimization (*PSO*), we chose 18 symmetrical complexes for which the atomistic structure has already been solved and that are available in the protein databank. As the evolutionary algorithms performing the search were stochastic, we repeated each test for 10 independent runs. Each execution could sample 20,000 candidate predictions before terminating. Information on the stoichiometry of the assemblies and the chosen interfacial spatial restraints can be found in the Supplementary Table S1.

As a general rule, a large number of interfacial spatial constraints leads to models of better quality. However, it has been observed that for our benchmarks set no more than three spatial constraints are necessary to efficiently guide the optimization process towards the assembly of realistic models^11^; hence the limitation of the number of spatial constraints generally to three per assembly prediction. In order to account for possible experimental noise on the target measures, an error of 2 Å was chosen per constraint, as previously done in ref. 11. Typical resolutions of macromolecular density maps range from 5 to 25 Å^25, 33, 34^; thus, an averaged resolution level of 15 Å was chosen for the evaluation mViE and *PSO*.

Evaluation of each method performance was achieved by computing the C*α*-RMSD between the already solved reference structures and the best representative structures of the candidate solutions, ranked by *ccc*. For each prediction problem, the top 5 ranking solutions were returned by each method as RMSD distances from the true assembly structure, resulting in 50 for each protein prediction problem per assembly protocol.

### Effect of spatial constraints on candidate solution assembly

With the aim to cover a broad spectrum of protein assembly cases, we chose 3 proteins complex of different size and stoichiometry. These were the lymphokine, GTP-cyclohydrolase and GroEL homo-multimers (Supplementary Fig. S3a,b **)**. These protein complexes were assembled under different conditions with the mViE protocol to assess how spatial constraints; both in quality and quantity, and density map resolution were affecting the quality of the candidate solutions. To address this task, 5 important contacting residues were identified within the interface of each of the 3 protein complexes and were chosen to serve as spatial restraints during optimization (Supplementary Fig. S3c **)**. For every optimization run the spatial constraints to be satisfied were randomly chosen in order to avoid bias toward preferred configurations and incremented gradually from 1 to 3 random restraints per run. To test for the effect of experimental error, the Euclidian distance for each of the spatial restraints was incremented from 2 to 6 Å. Finally, the density map resolution chosen to rank the best assemblies varied from 15 to 25 Å. In order to increase the significance of the statistics, 5 trials were performed for each condition. A total of 10,000 candidate predictions were generated per trial.

### Density map fitting as objective

Taking advantage of the fact that mViE does not require the computation of weights to balance the contribution of scoring function components, we directly used the *ccc* value during optimization instead of using it to rank the best models. Thus, for the three protein cases, the spatial as well as energetic terms of the traditional scoring function were used as inequality constraints on the search space. The *ccc* value was used as an objective to be maximized at each optimization step. Similarly to above, density map resolution and spatial constraint accuracy were varied from 15 to 25 Å and 2 to 6 Å respectively. In this case however, only one spatial constraint was randomly chosen for each optimization run, which was repeated in 5 trials.

In order to reduce computation time and keep the optimization in 4 dimensions [*α*, *β*, *γ*, *x*], the SITUS package was used every 1000 steps to align the input density map with the representative structures of the candidate models generated by mViE. In the normal optimization steps, the generated models were then aligned to the best SITUS fitted structure and the *ccc* value was computed. Blindly docking proteins inside density maps is a more difficult problem because the only information available consists of the density map of the fully assembled complex and the subunits structures. Thus for this problem, we did not use the residue contact information defined as spatial constraints as in the previous assembly cases. Instead, we used solely the information provided from the input density map of the complex at 15 Å and the energy potential as the 9-6 Lennard-Jones. In this case, the *ccc* was used as objective to be maximized and the energetic term as an inequality constraint (*E*_*phys*_ < 0). Moreover, we did not use the SITUS package to align the best structures to their input density map. Instead, any model generated was first translated to the center of the density map and assembled in 4 dimensions [*α*, *β*, *γ*, *x*]. In the same optimization step, the fully assembled complex was roto-translated in 6 dimensions in order to fit the complex inside the density map. This amounted to a total of 10 parameters to be optimized by mViE. Due to the inherent difficulty of the problem, a budget of 100,000 functions evaluations was used to blindly dock each of the three protein cases, with 5 trials per run.

### Discerning good from bad experimental spatial constraints

During the optimization driven assembly process, the presence of wrong spatial constraints in the constraints dataset may lead to incorrect models. It is therefore important to assess how the inclusion of wrong constraints can affect the quality of the predictions returned by assembly protocols. To this end, we chose three protein structures, which were the lymphokine, GTP-cyclohydrolase and GroEL homo-multimers, in order to test how the inclusion of erroneous spatial constraints into mViE affected the quality of solutions. For each protein case, a total of 5 correct and 5 wrong restraints were selected. The correct constraints corresponded to true contacts and the wrong ones, which corresponded to non-native residue contacts, were each selected randomly across the interacting proteins surface (Supplementary Table S4).

Both types of constraints were used by mViE to assemble protein models. Based on the assumption that wrong constraints should never outnumber corrects ones, they were gradually added from 1 to 4 in a random fashion together with correct ones. At the end of each run, models satisfying the highest numbers of constraints were selected and assessed for how good they were at discriminating good from bad constraints according to:

5 $$PPV=\frac{TP}{TP+FP}$$

where PPV is the precision score, TP and FP are the number of true positive and false positive satisfied constraints respectively. In this case a TP corresponds to a good constraint being correctly satisfied and FP to a wrong constraint being incorrectly satisfied in a candidate assembly. A total PPV score of 1.0 implies that the candidate model assembly satisfied only correct constraints and inversely for a score of 0.0.

### Assembly of the PhoQ homo-dimer

In order to reconstruct the physiological perisplasmic sensor homo-dimer of the PhoQ two-component signaling system (TCS) from a structural model, disulfide scanning mutagenesis experiments were undertaken by^27^. In these experiments, cysteine substitutions were performed on 16 residues spanning the N-terminal helix of the periplasmic region. For each of these residues, the degree of crosslinking was reported in Supplementary Table S5. Given that the degree of crosslinking roughly correlates with Cβ distance, this information could be used in the original study^27^ to reconstruct the physiological complex from the assembly subunits using a rigid-body grid search where translation and rotations are applied to one of the subunits while maintaining the other fixed. After generating several candidate assemblies, the best models were selected by their absence of steric clashes and by their Pearson correlation (*R*) value characterizing the correlation between the percentage of cross-linked residues curve, and Cβ distance from residues spanning the N-terminal helix of the models.

This problem was ideal to test our new protocol on a real biological case since it could be translated into a constrained optimization problem. Thus we used the same subunit model as used as in the study of^27^. Instead of using a grid search to sample the configuration of the homodimer interface, we used mViE. In the aim to get candidate assemblies resembling the physiological dimer, the Pearson value R between Cβ distance and fractional crosslinking Cβ distances was used as objective to be maximized upon satisfaction of the constraints. These were the Cβ distance between the most highly cross-linked residue (Arg50, crosslinking degree 0.95, Supplementary Table S5) and potential energy to avoid steric clashes. The best model was extracted and minimized using CHARMM27^35^. Our results were then compared with those obtained in ref. 27.

### Statistical analysis

Due to the ordinal and non-parametric nature of the RMSD values describing the quality of the best solutions returned by *PSO* and mViE, non-parametric statistical tests were used to evaluate significance of the results. In this paper, the Wilcoxon Rank Sum and Jonckheere-Terpstra tests were used.

## Electronic supplementary material

Supplementary Information
