# Supplementary material for: Disentangling constraints using viability evolution principles in integrative modeling of macromolecular assemblies
Source: Sci Rep. 2017 Mar 22;7:235. doi: 10.1038/s41598-017-00266-w (PMC5427971; doi:10.1038/s41598-017-00266-w)
Supplement: Supplementary file 1 — Supplementary Information [file 41598_2017_266_MOESM1_ESM.pdf]

Disentangling constraints using viability evolution principles in integrative modeling of macromolecular assemblies

Giorgio Tamò[1,2,#], Andrea Maesani[3,#], Sylvain Traeger[1,2], Matteo T. Degiacomi[4], Dario Floreano[3,*], Matteo Dal Peraro[1,2,*]

[1] Laboratory of Biomolecular Modeling, Institute of Bioengineering, École Polytechnique Fédérale de Lausanne, Lausanne, CH-1015, Switzerland

[2] Swiss Institute of Bioinformatics (SIB), Lausanne, CH-1015, Switzerland

[3] Laboratory of Intelligent Systems, Institute of Microengineering, École Polytechnique Fédérale de Lausanne, Lausanne, CH-1015, Switzerland

[4] Chemistry Research Laboratory, Department of Chemistry, University of Oxford, Oxford, UK

* Corresponding authors: matteo.dalperaro@epfl.ch, dario.floreano@epfl.ch

# These authors contributed equally.

Supplemental Information.

Table S1. Related to Figure 2; Protein prediction problems used to compare mViE and *PSO*.

Protein Name (PDBid)	Stoichiometry	Spatial restraint type	Target (+/- 2Å)
11S regulator (1avo)	7	resid 196 - 181	6
		resid 203 - 118	10
		resid 221 - 133	8
Acyl carrier (1fth)	3	width	60
		height	47
		resid 10 - 105	9
Alpha hemolysin (7ahl)	7	resid 2 - 56	11
		resid 162 - 35	6
		resid 128 - 131	7
Archeal sm (1i8f)	7	width	65
		height	37
		resid 29 - 29	4
Chaperonin (1h5x)	7	resid 95 - 7	4
		resid 61 - 57	12
		resid 56 - 55	10
chorismate mutase (1xho)	3	width	49
		height	44
		resid 74 - 74	4.5
Epimerase (1eq2)	5	resid 85 - 34	5
		resid 142 - 39	7
GP41 (1f23)	3	resid 26 - 46	8
		resid 11 - 63	15
		resid 2 - 71	9
Groel (1oel)	7	resid 518 - 37	9
		resid 257 - 269	5
		resid 283 - 181	5
GTP_cyclohydrolase (1fb1)	5	resid 241 - 243	9
		resid 224 - 134	6
		resid 183 - 126	7
lumazine synthase (1ejb)	5	width	77
		height	46
		resid 103 - 103	14
		resid 103 - 107	6
Lymphokine (1tnf)	3	resid 124 - 15	7
		resid 103 - 104	8
malporin sucrose (1af6)	3	resid 197 - 18	5
		resid 81 - 66	8
		resid 58 - 361	7
PA7 (1tzo)	7	resid 185 - 200	8
		resid 308 - 669	6
		resid 479 - 470	6
snRNP protein (1h64)	7	resid 22 - 65	10
		resid 5 - 41	6
tobacco virus (3kml)	17	height	32
		resid 13 - 13	33
		resid 25 - all	-26
trans regulator (1ny6)	7	resid 266 - 207	9
		resid 299 - 364	10
yjgF gene (1qu9)	3	resid 109 - 112	7
		resid 72 - 21	6

Table S2. Related to Figure 2; Evaluation of ranking methods using density map cross-correlation coefficient and energy potential. The relationship between ccc and energy to RMSD respectively was computed using Pearson's correlation. The r-value indicates the level of correlation between RMSD and ranking method, in this case a value of 1.0 indicates a perfect correlation. The superscripts indicate * P < 0.05, ** P < 0.01, *** P < 0.001 using a Pearson correlation test, *n.s.* no statistical significance.

	**ccc**		**energy**	
**protein predictions**	**r-val**	**p-val**	**r-val**	**p-val**
acyl_carrier	0.925	***	0.003	n.s
archael_sm	0.907	***	0.028	n.s
chorismate_mutase	0.072	n.s	0.132	n.s
groel	0.928	***	-0.01	n.s
GTP_cyclohydrolase	0.915	***	0.122	n.s
lumazine_syntase	0.869	***	0.003	n.s
malporin_sucrose	0.875	***	0.035	n.s
tobacco_virus	0.74	***	0.03	n.s
yjgF_gene	0.722	***	0.088	n.s
alpha_hemolysin	0.973	***	0.044	n.s
chaperonin	0.652	***	0.024	n.s
GP41	0.03	n.s	-0.081	n.s
lymphokine	0.803	***	0.023	n.s
PA7	0.913	***	0.026	n.s
snRNP_protein	0.764	***	0.042	*
11S_regulator	0.868	***	0.108	*
trans_regulator	0.649	***	0.037	*
epimerase	0.6812	***	0.0137	n.s

Table S3. Related to Figure 2; Performance comparison between mViE, PSO and other IM protocols. The best models extracted with mViE were compared to the best models obtained from an earlier implementation of *pow^{er}* [a], SymmDock[b] and Multifit[c] as extracted from the literature. Values in bold format indicate the assembly protocol that obtained the most native resembling models.

	**Best Models RMSDs (Å-Cα)**			
**protein predictions**	***mVie***	***PSO***	**Literature**	(ref)
acyl carrier	**1.50**	**1.50**	1.91	a
archeal SM	1.09	1.34	**0.95**	a
chorismate mutase	**1.49**	1.61	1.52	a
groel	**1.86**	1.89	2.76	b
GTP cyclohydrolase	1.01	**0.94**	1.24	b
lumazine synthase	**1.60**	1.72	1.89	a
tobacco virus	**0.95**	1.17	-	-
malporin sucrose	**0.64**	7.23	1.21	b
ygjf gene	**1.27**	1.45	2.03	b
alpha hemolysin	**0.75**	1.05	2.15	c
chaperonin	**0.57**	0.74	2.25	b
GP41	2.40	2.75	**0.95**	b
lymphokine	**1.41**	1.45	1.62	b
PA7	**0.84**	0.86	3.17	b
snRNP protein	**0.70**	0.91	3.44	b
11S regulator	1.92	1.89	**0.63**	b
trans regulator	2.34	2.67	**1.36**	b
epimerase	**1.56**	2	2.88	b

Table S4. Related to Figure 3; True and False spatial constraints selected for the Lymphokine, GTP-cyclohydrolase and GroEL assembly cases. Carbon-α distances between the residues of the subunits are expressed in Å.

	**True restraints**			**False restraints**		
**protein name**	**Subunit 1**	**Subunit 2**	**Distance[Å]**	**Subunit 1**	**Subunit 2**	**Distance[Å]**
Lymphokine	Phe124	His15	7	Arg31	Ala145	36
	Glu103	Arg104	8	Gln102	Asn34	33
	Glu116	Lys98	13	Lys98	His15	15
	Lys98	Glu116	6	Gly54	Val150	22
	Tyr119	Tyr119	8	Gly122	Lys112	30
GTP Cyclo.	Lys220	Asp136	6	Ser228	Ser166	18
	Glu183	His126	7	Pro238	Phe122	20
	Lys224	Asp134	6	Leu82	Glu61	30
	Leu245	Leu247	7	Arg216	Lys93	32
	Arg241	Glu243	9	Als208	Glu243	18
GroEL	Glu518	Asn37	9	Gly256	Phe44	14
	Glu257	Lys272	11	Asp5	Lys34	24
	Glu255	Lys207	8	Asp359	Gly459	46
	Arg197	Glu386	14	Glu255	Val387	19
	Asp283	Thr181	5	Glu76	Glu209	22

Table S5. Related to Materials and Methods section; Experimental crosslinking data adapted from residues 47 to 62 spanning the N-terminal helix of the periplasmic sensor domain 3BQ8. The theoretical values below are adapted computed using equation [1] found in the work by Goldberg et al. 2008.

	Degree of Crosslinking	
**Residue**	**Experimental**	**Theoretical**
T47	0.69	0.84
T48	0.38	0.57
F49	0.32	0.31
R50	0.95	0.75
L51	0.92	0.72
L52	0.81	0.3
R53	0.5	0.61
G54	0.85	0.82
E55	0.09	0.37
S56	0.34	0.46
N57	0.81	0.85
L58	0.71	0.5
F59	0.28	0.34
Y60	0.92	0.8
T61	0.48	0.65
L62	0.12	0.29

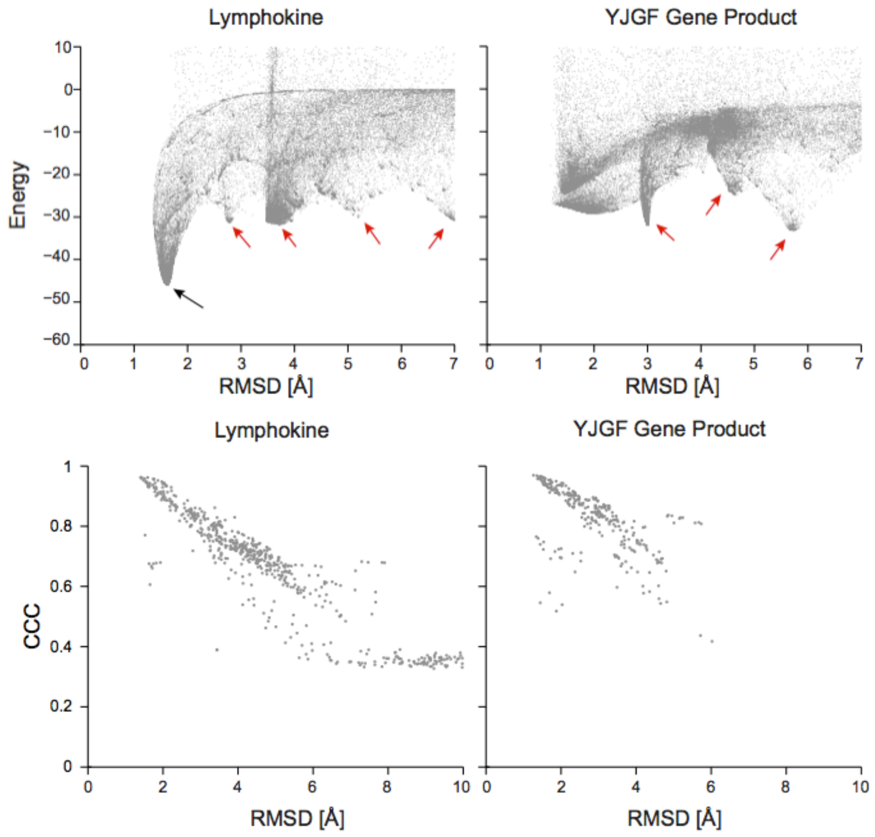

Figure S1. Related to Figure 2; Assessment of the effect of ccc/energy on backbone RMSD of the model assemblies. (Top Panel) The symmetrical assembly models of the Lymphokine and YJGF gene product were evaluated for a relationship between backbone RMSD and energy as defined by a Lennard-Jones potential. Following the energy gradient may help reaching search areas were assemblies having minimal RMSD are found (black arrow). However, the presence of energy wells can mislead an optimization method (red arrows). In some cases, as in the YJGF gene product, this is even more troublesome as energy wells corresponding to configurations with higher RMSD have the lowest energy. **(Bottom Panel)** In contrast, there was for the same assembly cases a more correlating relationship between RMSD and cross-correlation-coefficient computed against Cryo-EM volumetric maps.

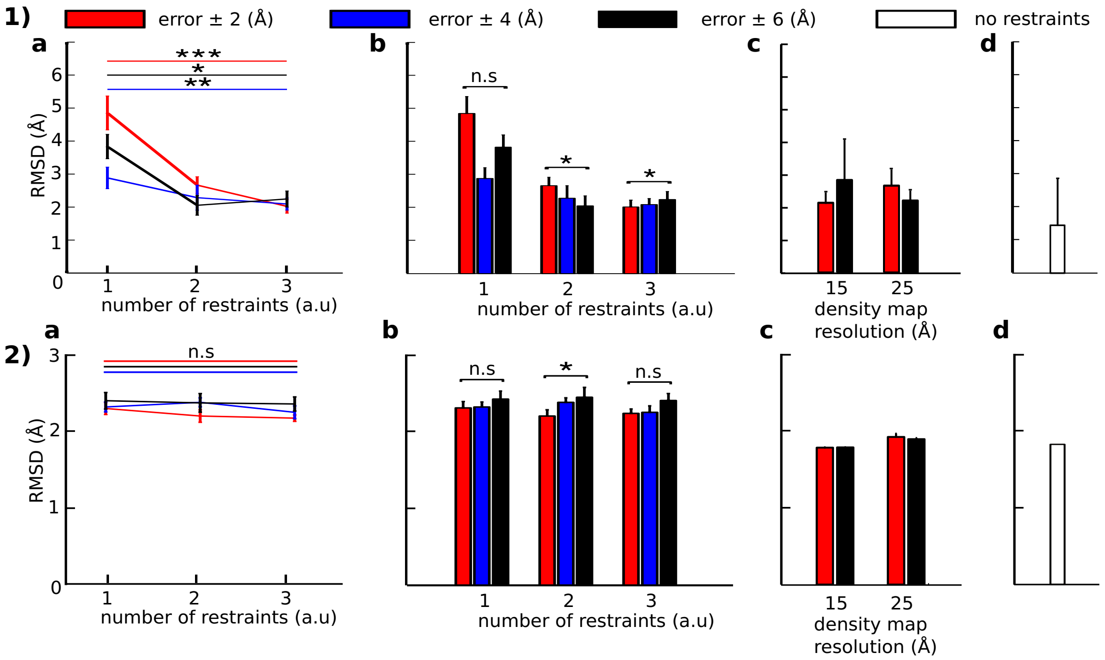

Figure S2. Related to Figure 2; Performance assessment of mViE on the GroEL (1) and Lymphokine (2) assembly cases. a. Effect of number of geometric spatial constraints on the quality of candidate assemblies returned by mViE. Here the spatial constraints and potential energy were used as constraints on the search space, population diversity as objective and density map ranking as a postprocessing step. For the assembly cases of GroEL, increasing the number of spatial restraints had a more significant and drastic effect on the quality of constraints than in the case of the Lymphokine case **b**. Effect of the quality of spatial constraints (measured as error ± Å) on the quality of candidate assemblies returned by mViE. The assembly condition were the same as in **a**. In this case of Lymphokine and GroEL, increasing the quality of spatial restraints had little effect on the quality of the assemblies returned by mViE **c.** Results of mViE protocol using density map fitting during assembly of GroEl (1) and Lymphokine (2). The optimization was undertaken by using *ccc* as an objective to be maximized once the geometry and energy constraints are satisfied. **d.** Blind docking using mViE on the GroEL and Lymphokine assembly problems. As input were provided a density map of 15 Å and one of the multimer subunits. The *ccc* was used as objective to be maximized during the optimization. Only the 9-6 Lennard Jones energy potential was used as constraint.

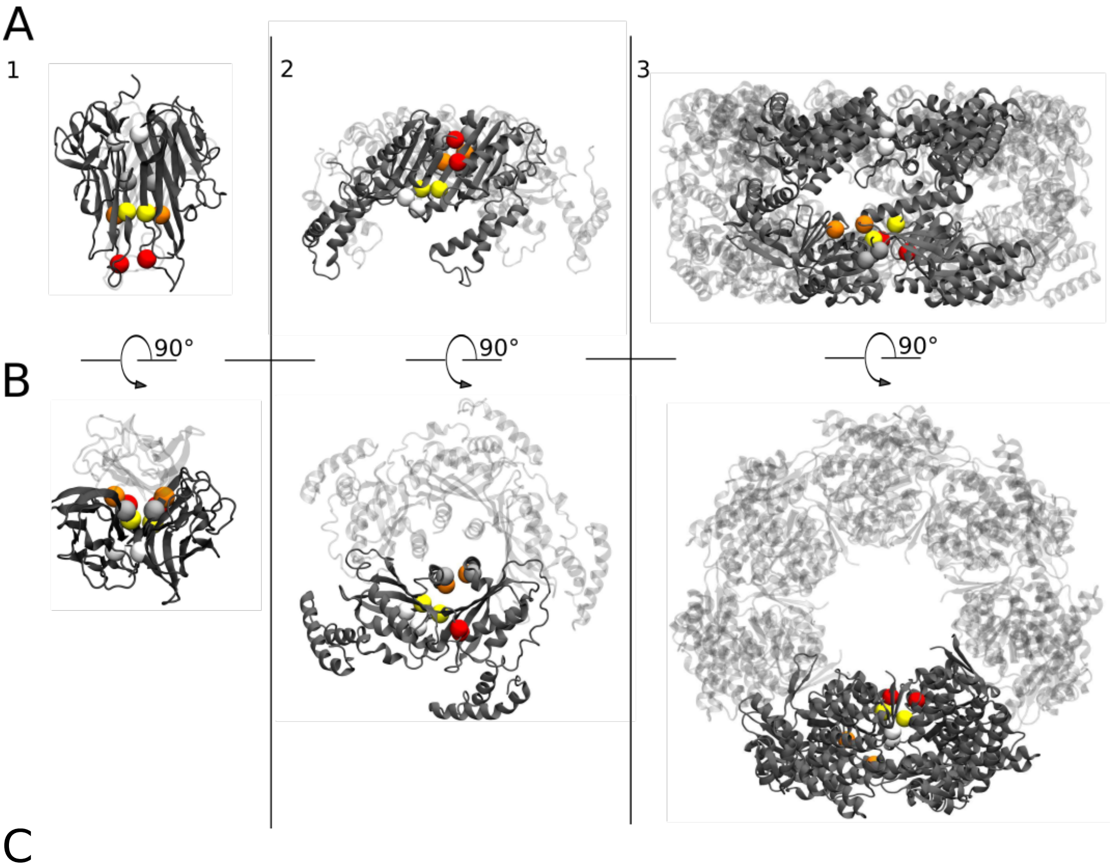

C

Protein Name	Interaction Color	Interacting residue ids		Interacting residue name	
		subunit 1	subunit 2	subunit 1	subunit 2
Lymphokine [pdb 1tnf]	white	124	15	Phe	His
	red	103	104	Glu	Arg
	yellow	116	98	Glu	Lys
	orange	98	116	Lys	Glu
	gray	119	119	Tyr	Tyr
GTP cyclo. [pdb 1fb1]	white	220	136	Lys	Asp
	red	183	126	Glu	His
	yellow	224	134	Lys	Asp
	orange	245	247	Leu	Leu
	gray	241	243	Arg	Glu
Groel [pdb 1oel]	white	518	37	Glu	Asn
	red	257	272	Glu	Lys
	yellow	255	207	Glu	Lys
	orange	197	386	Arg	Glu
	gray	283	181	Asp	Thr

Figure S3. Related to Figure 2; Circular assembly cases used for the performance assessment of mViE. A-B. Assembly cases of the Lymphokine (1), GTP-Cyclohydrolase (2) and GroEL (3). **C.** Important interfacial residues chosen as spatial restraints during the assessment performance of mViE and corresponding color codes (See Methods).
